# Basic and associated causes of schistosomiasis-related mortality in Brazil: A population-based study and a 20-year time series of a disease still neglected

**DOI:** 10.7189/jogh.11.04061

**Published:** 2021-10-09

**Authors:** Wandklebson Silva da Paz, Erica dos Santos Reis, Iane Brito Leal, Yanna Menezes Barbosa, Karina Conceição GM de Araújo, Amélia Ribeiro de Jesus, Carlos Dornels Freire de Souza, Allan Dantas dos Santos, Márcio Bezerra-Santos

**Affiliations:** 1 Parasitic Biology Graduate Program, Universidade Federal de Sergipe, Aracaju, Sergipe, Brazil; 2 Tropical Medicine Graduate Program, Universidade Federal de Pernambuco, Recife, Pernambuco, Brazil; 3 Health Sciences Graduate Program, Universidade Federal de Sergipe, Aracaju, Sergipe, Brazil; 4 Department of Morphology, Universidade Federal de Sergipe, Aracaju, Sergipe, Brazil; 5 Department of Medicine, Universidade Federal de Sergipe, Aracaju, Sergipe, Brazil; 6 Immunology and Molecular Biology Laboratory, University Hospital, Universidade Federal de Sergipe, Aracaju, Brazil; 7 Department of Medicine, Universidade Federal de Alagoas, Arapiraca, Alagoas, Brazil; 8 Department of Nursing, Universidade Federal de Sergipe, Lagarto, Sergipe, Brazil; 9 Nursing Graduate Program, Universidade Federal de Sergipe, Aracaju, Sergipe, Brazil

## Abstract

**Background:**

Schistosomiasis is a persistent public health problem in Brazil. Regardless advances in diagnosis and mass treatment, schistosomiasis has a severe impact on morbimortality in the country and remains a neglected tropical disease. Herein, we assessed the basic and associated causes of schistosomiasis-related deaths and the temporal and spatial patterns of mortality from the disease in Brazil between 1999 and 2018.

**Methods:**

We conducted an ecological and time series study. The segmented log-linear regression model was applied to assess time trends, considering all deaths recorded in the category B65/ICD-10. Additionally, we elaborated maps of mortality rates from schistosomiasis in Brazil.

**Results:**

A total of 4168 schistosomiasis-related deaths were recorded in Brazil in this period, as an associated cause. Time trend analysis revealed an increase in the average age of deaths from schistosomiasis (annual percentage change (APC) = 0.84), and stable trend in Brazil (APC = 0.31). Concerning schistosomiasis-related deaths, we observed disorders related to the digestive system, liver diseases, septicemias, and chronic diseases. Surprisingly, there were deaths caused by non-endemic *Schistosoma* species in Brazil. Also, municipalities from non-endemic areas in Brazil presented schistosomiasis-related deaths.

**Conclusion:**

Altogether, our analyses demonstrated that schistosomiasis remains a significant cause of death in Brazil, and it is increasing in some areas, especially in the Northeast region. Additionally, women and the elderly showed a stable time trend of deaths. Thereby, it urgently requires improvements in the control programs strategies, in the sense of an effective reduction in cases and deaths from the disease in Brazil.

Schistosomiasis is the second most impacting parasitic disease in the world (behind only malaria), with about 240 million cases. It is considered a neglected tropical disease and affects mainly areas of greatest social vulnerability in sub-Saharan Africa, Asia, and Latin America [1]. Brazil is endemic only for the species Schistosoma mansoni, which causes intestinal schistosomiasis. The disease has two clinical phases: the acute phase eventually presents cercarial dermatitis and non-specific symptoms such as fever, nausea, and headache; in the chronic phase there may be abdominal pain, diarrhea, and blood in the stool [1]. Liver and spleen enlargement is observed in advanced cases and is frequently associated with portal hypertension, ascites, collateral circulation, and esophageal varices. The severe hepatosplenic form is responsible for most deaths in schistosomiasis [1,2]. To date, praziquantel is the recommended treatment against all clinical forms of the diseases. This drug is safe, effective, low-cost, and reduce the risk of developing the severe clinical forms and deaths from schistosomiasis [1].

In Brazil, by virtue of the activities of the Schistosomiasis Control Program (PCE), there has been a significant reduction in schistosomiasis severity indicators in recent decades [3]. Additionally, the improvements in health service strategies, as diagnosis and mass treatment in endemic areas, have dropped the number of cases and deaths and increased life expectancy for ill patients [3]. Regardless all these measures, schistosomiasis remains endemic in many states, mainly in the Northeast region [4,5]. PCE estimates point to 1.5 million individuals infected with *S. mansoni* in Brazil. More importantly, many of these cases occur in the elderly, and this considerably increases the risk of association with chronic non-infectious diseases as cardiovascular or digestive diseases, and neoplasms. Furthermore, prior studies conducted by our group showed an increase in the schistosomiasis mortality rate in age groups above 60 years [4,6]. Thereby, it suggests the chronicity of the disease, a higher risk of association with comorbidities, and death [3,7].

The World Health Organization (WHO) recommends that mortality statistics should be presented according to the basic cause of death, defined as the disease or injury that initiated the sequence of events that directly caused death [8-10]. Notwithstanding, especially for infectious and parasitic diseases, there is also a need for more comprehensive information related to death [8,9]. Herewith, it is necessary to consider all mortality causes recorded in the death certificates that include, in addition to the basic causes, the associated causes.

Notably, the occurrence of comorbidities increases the risk of clinical complications and deaths from schistosomiasis and this can be evaluated by identifying associated causes in the death certificate [9]. Considering this, assessing the basic and associated causes of death can improve to health systems, especially in mortality risk analysis. In that regard, temporal and spatial analysis techniques are one of the most efficient methods, since they allow to monitor the impacts of the disease over time and in space [9,11]. Several studies have used spatial and spatiotemporal analysis tools to comprehend the dynamics of schistosomiasis in Brazil [4,6,12]. Nevertheless, there are no studies investigating the associated causes of death from the disease at the national level. Herein, we hypothesize that the mortality rate from schistosomiasis may be higher among men, as they are usually more affected by the disease in endemic areas. Also, mortality from schistosomiasis is higher in the elderly, especially among those with some coexisting illnesses.

Considering that *S. mansoni* infection remains an important public health concern in Brazil and the lack of studies assessing comorbidities related to mortality from schistosomiasis, we conducted the first study to assess the basic and associated causes, and the temporal and spatial patterns of schistosomiasis-related deaths in Brazil between 1999 and 2018.

## METHODS

### Type of study and period

We conducted an ecological, population-based, and time series study (from 1999 to 2018), using temporal and spatial analysis tools. We delimited this period considering that since 1999 Brazil started to use a new version of the Mortality Information System (SIM), for which a new death certificate was established [13]. After that, there were improvements in data recording, with more detailed filling in of the death certificate and inclusion of additional information, as associated causes of death [8].

### Study area

Brazil is in South America and is the fifth largest country in the world, with approximately 211 million inhabitants [14]. The country is divided into 27 federative units and 5570 municipalities. In addition, the states are grouped into five regions: North, Northeast, Southeast, South, and Midwest, with distinct geographical, economic, and cultural characteristics. In physiographic aspects, Brazil has an extensive area of tropical forests, as the Amazon and Atlantic rainforest, and extensive water collections throughout the territory [14]. Regardless being classified as the 12th largest economy in the world (Gross Domestic Product - GDP/2020 = R$7.4 trillion; dropped 4.1% last year), Brazil has important socioeconomic disparities [14]. Furthermore, there is a lack of a sewerage system and safe-drinking water access in many municipalities, which maintains the schistosomiasis transmission cycle [6,15].

### Data source

Data was obtained from the death certificate, available in the SIM of the Brazilian Ministry of Health. This statement is the standard SIM document and consists of a form filled out by medical professionals and contains demographic and clinical information, regarding the basic and associated causes of death [16]. SIM data are in public domain and can be obtained from the website of the Informatics Department of the Unified Health System (DATASUS). Additionally, population data were obtained from the Brazilian Institute of Geography and Statistics (IBGE), based on data from the national population census in 2000 and 2010, and official estimates for the inter-census years. The digital cartographic mesh, in shapefile format, was extracted from the Geographic Projection System, of the IBGE website (Geodesic Reference System, SIRGAS-2000).

### Variables and measures

Herein, we assessed the following variables:

- Absolute number of schistosomiasis-related deaths (considering basic and associated causes) registered in the 5570 municipalities;
- Crude mortality rates for schistosomiasis-related deaths. Rates were calculated dividing the number of deaths from schistosomiasis by the population. The result was multiplied by 100 000 inhabitants in each municipality, state, and region. These rates were also calculated according to gender and age group;
- Average age at death for each year of the study;
- Proportion between the number of municipalities with death records and the total of municipalities for each year.

### Data analysis

#### Evaluation of the epidemiological profile of schistosomiasis-related deaths in Brazil

Deaths from schistosomiasis are included in the subcategories from the category B65 (Schistosomiasis-Bilharziasis), from the International Statistical Classification of Diseases and Related Health Problems, 10th Revision (International Classification of Disease/ICD-10) [17]. After selecting deaths from schistosomiasis as an associated cause, we performed the descriptive epidemiological characterization of these deaths. We carried out a bivariate statistical analysis of the factors associated with deaths from schistosomiasis. For variables with only two categories, they were compared to each other (ie, gender). For variables with more than two categories, each one was compared with the one with the lowest number of deaths (ie., age group). We also calculated the odds ratio (OR) and the 95% confidence intervals (95% CI) for all assessed variables. For these statistical analyses, we used the χ^2^ test, and the results were considered statistically significant when *P*-value <0.05 was obtained.

### Description of diseases and disorders associated with mortality from schistosomiasis in Brazil

Subsequently, we conducted the decoding and description of the basic and associated causes, in which schistosomiasis was mentioned, from the death certificates:

- we include all associated causes mentioned in the death certificates, in which schistosomiasis was considered the basic cause of death;
- correspondingly, we considered all the basic causes of death, in which schistosomiasis was mentioned as an associated cause.

Usually in death certificates, they repeat the same basic cause among the associated causes of death [18]. Hereupon, to avoid repetition bias in the description of associated causes, mention that also repeated schistosomiasis as the basic cause was excluded. Concerning that, tables were created with data on the basic and associated causes of deaths from schistosomiasis. We organized them in a ranking of absolute and percentage frequencies.

#### Time trend analysis

Next, we assessed the time trends of the data using joinpoint regression models (segmented linear regression). This method allows to verify changes in the trend of a variable over time [19]. First, we used the Monte Carlo permutation test to identify the best segment of each model. We applied 9999 permutations and the best model was the one with the highest residue determination coefficient (R2). Afterwards, the annual percentage change (APC) and its respective confidence interval (95% CI) were calculated for each segmented period. Herein, positive and significant APC (*P*-value <0.05) indicates an increasing time trend. Alternatively, negative and significant APC indicates a decreasing trend, while APC that was not significantly different, indicates a stationary trend [19]. In addition, when a time trend had inflection points and more than one APC, we calculated the average annual percentage change (AAPC) for the entire period. The time trends were considered statistically significant when APC or AAPC had a *P*-value <0.05 and their 95% CI does not include a zero value.

#### Spatial distribution of data

Lastly, we elaborated choropleth maps of Brazil, divided by municipalities, representing the schistosomiasis-related deaths, and considering the basic or associated cause of death, available in subcategory B65/ICD-10. Additionally, data and maps were stratified according to the following categories: *Schistosoma haematobium* (B65.0); *Schistosoma mansoni* (B65.1); *Schistosoma japonicum* (B65.2); Cercarial dermatitis (B65.3); Other schistosomiases (B65.8); Schistosomiasis non-specified (B65.9).

### Software

The study data were tabulated in Microsoft Excel (2019) spreadsheets. Statistical analysis was performed using the GraphPad Prism version 8.0.1. For time trend analysis, we used the Joinpoint Regression Program version 4.2.0. For spatial analysis and construction of choropleth maps, we used the QGis software version 3.4.

## RESULTS

We identified a total of 4168 deaths from schistosomiasis, as an associated cause, in Brazil between 1999 to 2018. Considering the epidemiological variables of the study, the highest OR for schistosomiasis-related deaths were observed in women, in the age groups of 40-59 and ≥60 years old, and in patients from the Northeast and Southeast regions (**Table 1**). Moreover, we compared the total number of deaths among the three states with the highest proportions of deaths from schistosomiasis and the other states of the country (Table S1 in the **Online Supplementary Document**) and interestingly, we observed that patients from these three states together showed about 4-fold higher chance of death from schistosomiasis in relation to all other states in Brazil.

**Table 1:** Bivariate statistical analysis between epidemiological variables and deaths related to schistosomiasis as an associated cause in Brazil, between 1999 and 2018

Variables*	Schistosomiasis-related deaths^†^					
**Yes** (n)	**No** (n)	**%**	**OR**	**95% CI**	***P*-value**
**Gender:**						
Male	2216	12 712 845	0.017	0.84	0.79 to 0.90	-
Female	1952	9 516 064	0.021	1.17	1.10 to 1.25	<0.0001
**Age group:**						
0-9 y	16	1 192 053	0.001	-		-
10-19 y	35	510 019	0.007	5.11	2.83 to 9.23	<0.0001
20-39 y	319	2 328 500	0.014	10.2	6.17 to 16.86	<0.0001
40-59 y	1316	4 492 863	0.029	21.8	13.33 to 35.73	<0.0001
≥60 y	2482	13 635 269	0.018	13.5	8.29 to 22.17	<0.0001
**Region of Brazil:**						
North	62	1 267 876	0.002	-		-
Northeast	6746	5 673 787	0.045	24.9	16.57 to 37.67	<0.0001
Southeast	3196	10 412 723	0.014	7.94	5.26 to 11.99	<0.0001
South	73	3 487 799	0.001	0.37	0.21 to 0.67	<0.0001
Midwest	174	1 395 344	0.003	1.85	1.12 to 3.05	0.013
**Federative units:**						
Other states	1362	15 574 605	0.008	0.20	0.19 to 0.22	-
PE, SP, and AL	2806	6 667 087	0.042	4.81	4.51 to 5.13	<0.0001

n – number of samples, OR – odds ratio, CI – confidence interval, SP – São Paulo, PE – Pernambuco, AL – Alagoas

*We excluded missing and/or ignored data, or lacking information from the municipality of residence.

†Deaths related to schistosomiasis as an associated cause in Brazil.

Concerning the temporal patterns of schistosomiasis mortality as an associated cause, we observed a stable time trend in Brazil between 1999 and 2018 **(****Table 2****)**. Likewise, we observed a stable time trend in all regions of the country. However, the Northeast region showed an increasing time trend in deaths from schistosomiasis between 1999 and 2016. Correspondingly, there was an increasing trend of deaths among women in the same period. On the other hand, there was stability in deaths among men. Despite the decreasing trend in most age groups, we observed stability in deaths among patients ≥60 years old. Conversely, we identified an increasing time trend in the average age at death from schistosomiasis.

**Table 2:** Temporal trend analyses of schistosomiasis-related deaths as an associated cause, according to sociodemographic variables and places of residence in Brazil from 1999 to 2018

Variables	Period	APC	95% CI	Time Trend	*P*-value
**Brazil:**					
Associated cause	1999-2018	0.31	-0.72 to 0.90	Stable	0.855
**Regions:**					
North	1999-2018	-3.02	-10.03 to 0.52	Stable	0.072
Northeast†	1999-2016	1.52*	0.20 to 2.91	Increasing	0.029
	2016-2018	-12.22	39.71 to 27.74	Stable	0.469
Southeast	1999-2018	-1.10	-2.31 to 0.22	Stable	0.097
South	1999-2018	-1.50	-4.71 to 1.90	Stable	0.367
Midwest	1999-2018	-3.13	-7.13 to 1.03	Stable	0.130
**Proportion of municipalities with deaths:**	1999-2018	-0.03*	-0.01 to 0.02	Decreasing	<0.0001
**Gender**					
Male	1999-2018	-0.53	-1.54 to 0.51	Stable	0.308
Female‡	1999-2016	1.33*	0.65 to 2.04	Increasing	0.001
	2016-2018	-14.10	-29.55 to 4.51	Stable	0.119
**Age group (years):**					
0 - 9	1999-2018	-0.45	-3.23 to 2.54	Stable	0.781
10-19	1999-2018	1.44	-4.44 to 7.54	Stable	0.632
20-39	1999-2018	-6.70*	-8.53 to -4.91	Decreasing	<0.0001
40-59	1999-2018	-4.01*	-5.23 to -2.72	Decreasing	<0.0001
≥60	1999-2018	-0.41	-1.22 to 0.52	Stable	0.377
**Average age at death**	1999-2018	0.84*	0.74 to 0.95	Increasing	<0.0001

APC – annual percentage change, AAPC – average annual percentage change, CI – confidence interval

*P-value <0.05.

†Northeast region AAPC = -0.01; 95% CI = -3.73 to 3.86; *P-*value = 0.99.

‡Female AAPC = -0.5; 95% CI = -2.4 to 1.5; *P-*value = 0.66.

Interestingly, when we considered all the associated causes to deaths from schistosomiasis, we identify as the main causes those regarding to the digestive system, mainly disorders involving hepatic impairment (**Table 3**). The highest percentages were observed in unspecified diseases of the digestive system (K92 = 13.51%), unspecified liver diseases (K76 = 11.28%), shock (R57 = 8.90%), esophageal varices (I85 = 8.10%), liver fibrosis and cirrhosis (K74 = 7.85%), and liver failure (K72 = 7.79%). Similarly, when we consider the basic causes of death, in which schistosomiasis was included as an associated cause, we identified mainly disorders related to the digestive system (31.02%), especially liver diseases (**Table 4**). It was found that the main basic causes were concentrated in the chapters on diseases of the digestive system, mainly involving the liver, which together reached 31.02% of deaths from schistosomiasis as an associated cause. In addition, infectious diseases such as hepatitis were present in 6% of deaths as basic cause.

**Table 3:** Identification and frequency (absolute and percentage) of associated causes with deaths from schistosomiasis in Brazil between 1999 and 2018

Associated causes with deaths from schistosomiasis (ICD-10)	n	%
Other diseases of the digestive system (K92)	3,873	13.51
Other liver diseases (K76)	3234	11.28
Shock not elsewhere classified (R57)	2550	8.90
Esophageal varices (I85)	2321	8.10
Liver fibrosis and cirrhosis (K74)	2251	7.85
Hepatic impairment, not elsewhere classified (K72)	2234	7.79
Other septicemias (A41)	1179	4.11
Other general symptoms and signs (R68)	974	3.40
Respiratory failure, not elsewhere classified (J96)	711	2.48
Other symptoms and signs related to the circulatory and respiratory systems (R09)	632	2.20
Essential hypertension (primary) (I10)	489	1.71
Diabetes mellitus (E14)	442	1.54
Peritonitis (K65)	434	1.51
Ascites (R18)	423	1.48
Unspecified pathogen pneumonia (J18)	405	1.41
Heart failure (I50)	348	1.21
Acute renal failure (N17)	315	1.10
Other anemias (D64)	310	1.08
Other respiratory disorders (J98)	252	0.88
Unspecified renal failure (N19)	229	0.80
Abnormal patient reaction or late complication, caused by surgical intervention and other surgical acts, with no mention of accident during the intervention (Y83)	200	0.70
Other forms of pulmonary heart disease (I27)	196	0.67
Unspecified pulmonary edema (J81)	187	0.65
Chronic renal failure (N18)	168	0.59
Unspecified protein-calorie malnutrition (E46)	163	0.57
Mental and behavioral disorders due to alcohol use (F10)	162	0.57
Hepatomegaly and splenomegaly, not elsewhere classified (R16)	146	0.51
Hemorrhage, not elsewhere classified (R58)	146	0.51
Other disorders of hydroelectrolytic and acid-base balance (E87)	139	0.48
Acute post-hemorrhagic anemia (D62)	120	0.42
Complications of heart disease and undefined heart disease (I51)	110	0.38
Other associated causes of death	3321	11.59
**Total**	**28** **664**	**100.00**

**Table 4:** Identification and frequency (absolute and percentage) of the basic causes of death, in which schistosomiasis was mentioned as an associated cause in Brazil between 1999 and 2018

Basic causes of schistosomiasis-related deaths (ICD-10)	n	%
Alcoholic liver disease (K70)	305	7.32
Malignant neoplasm of the liver and intrahepatic bile ducts (C22)	237	5.69
Unspecified pathogen pneumonia (J18)	224	5.37
Diabetes mellitus (E14)	197	4.73
Acute myocardial infarction (I21)	163	3.91
Essential hypertension (I10)	123	2.95
Other liver diseases (K76)	114	2.74
Chronic viral hepatitis (B18)	108	2.59
Cardiomyopathies (I42)	93	2.23
Hypertensive heart disease (I11)	90	2.16
Hepatic impairment, not elsewhere classified (K72)	81	1.94
Other septicemias (A41)	79	1.90
Other respiratory disorders (J98)	78	1.87
Heart Failure (I50)	61	1.46
Liver fibrosis and cirrhosis (K74)	58	1.39
Other urinary tract disorders (N39)	51	1.22
Acute hepatitis B (B16)	50	1.20
Peritonitis (K65)	50	1.20
Chagas disease (B57)	48	1.15
Chronic ischemic heart disease (I25)	48	1.15
Other chronic obstructive pulmonary diseases (J44)	46	1.10
Other forms of pulmonary heart disease (I27)	40	0.96
Diarrhea and gastroenteritis of presumed infectious origin (A09)	39	0.94
Complications of heart defects and undefined heart diseases (I51)	32	0.77
Stroke, not specified as hemorrhagic or ischemic (I64)	32	0.77
Malignant neoplasm of stomach (C16)	31	0.74
Vascular disorders of the intestine (K55)	31	0.74
Other cerebrovascular diseases (I67)	30	0.72
Other acute viral hepatitis (B17)	29	0.70
Intracerebral hemorrhage (I61)	29	0.70
Cholelithiasis (K80)	28	0.67
Other basic causes of death	1543	37.02
**Total**	**4168**	**100.00**

**Table 5** shows the frequency of deaths according to the clinical forms of schistosomiasis and included in the category B65 of death certificates. We observed that the main clinical form of deaths was intestinal schistosomiasis, among the basic and associated cause. Surprisingly, although Brazil has no record of transmission of other *Schistosoma* species, we identified deaths from schistosomiasis caused by *Schistosoma haematobium* and *Schistosoma japonicum*. Unfortunately, there was no specification of the type of schistosomiasis, or the parasite, in 32.22% of the basic and in 42.97% of the associated causes.

**Table 5:** Absolute frequency and percentage of deaths from schistosomiasis, according to the clinical presentation of the ICD-10 (category B65) in the death certificate, among the basic and associated causes in Brazil, from 1999 to 2018

Clinical presentation of schistosomiasis-related deaths and *Schistosoma* species (ICD-10)	Basic causes		Associated causes	
**n**	**%**	**n**	**%**
Schistosomiasis caused by *Schistosoma mansoni* (intestinal schistosomiasis – B65.1)	6210	60.58	2,185	52.42
Schistosomiasis caused by *Schistosoma haematobium* (urinary schistosomiasis – B65.0)	211	2.06	20	0.48
Schistosomiasis caused by *Schistosoma japonicum* (intestinal schistosomiasis – B65.2)	24	0.23	14	0.34
Cercarial dermatitis (B65.3)	1	0.01	2	0.05
Another schistosomiasis (B65.8)	400	3.90	156	3.74
Unspecified schistosomiasis (B65.9)	3405	33.22	1791	42.97
**Total**	**10** **251**	**100.00**	**4168**	**100.00**

ICD – International Classification of Disease

**Figure 1:**
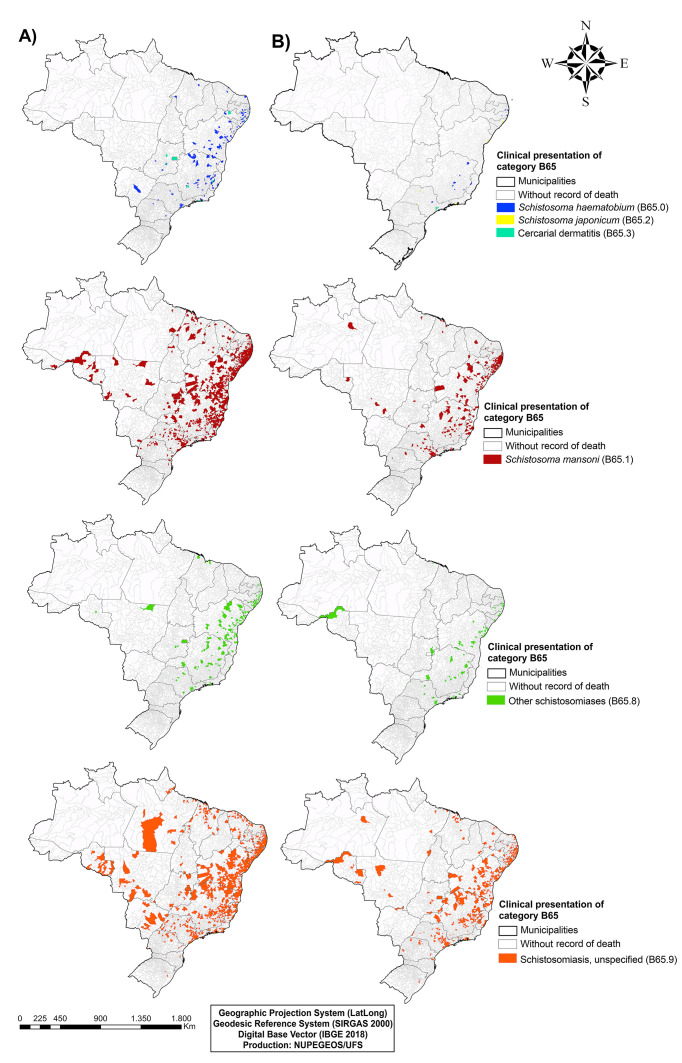
Spatial distribution maps of deaths from schistosomiasis in Brazil, according to the disease’s clinical presentation of the ICD-10. **A)** Basic causes of death. **B)** Associated causes of death.

Spatial analysis maps showed that deaths were concentrated in municipalities located on the country's coastal strip, mostly in states from the Northeast and Southeast regions (**Figure 1****,** Panel A and B). Surprisingly, although the North region is considered non-endemic for schistosomiasis, data on spatial distribution showed records of deaths in municipalities from that region.

Additionally, when assessing the distribution of deaths from basic causes, most municipalities had unspecified schistosomiasis as the main cause of death (n = 965 municipalities), followed by schistosomiasis caused by *S. mansoni* (n = 922; **Figure 1****,** Panel A). Interestingly, there was record of death from urinary schistosomiasis (by *S. haematobium*) in 145 municipalities, and from *S. japonicum* infection in 22 municipalities. Likewise, when considering the associated causes, the main clinical form was unspecified schistosomiasis (n = 541; **Figure 1****,** Panel B), followed by schistosomiasis by *S. mansoni* (n = 363). Furthermore, there were records of deaths from urinary schistosomiasis (n = 18) and from *S. japonicum* infection (n = 14).

## DISCUSSION

Despite all the measures implemented by the PCE, as diagnosis and mass treatment of populations in endemic areas, schistosomiasis still has a high number of cases and deaths, and remains a serious public health concern in Brazil^[^6,11,20^]^. Herein, we conducted a pioneer, and population-based study, to assess the epidemiological profile and spatial and temporal patterns of deaths from schistosomiasis in Brazil, as basic and associated cause, in a period of 20 years. Importantly, our analyzes demonstrate stability of deaths from the disease in the country during the study period. Furthermore, we identified the occurrence of deaths in non-endemic municipalities and deaths caused by non-endemic *Schistosoma* species in Brazil. Taken together, our analyses allowed us to understand more reliably the dynamics of deaths from the disease in Brazil over time and in space.

Surprisingly, we observed higher schistosomiasis mortality rates among women. Usually, men are more exposed to *S. mansoni* infection, due to occupational activities (ie, fishing or farming), or leisure activities [6,21,22]. As a result, schistosomiasis death use to be higher in this group [4,6,23]. On the other hand, we observed a decreasing time trend in the mortality rate in men, and stability of deaths in women. We consider that most of cases among women occur in riverside communities from endemic areas. Despite the actions of the PCE, there was no control of the disease in these regions over time and, therefore, maintained the transmission and stability of deaths among women.

Time trend analyzes also showed stability in deaths from schistosomiasis in all regions of Brazil. However, the Northeast region showed an increasing trend from 1999 to 2016. Notably, Brazil is a country of continental dimensions and with significant physiographic and socioeconomic differences among regions. Thereby, these regional disparities and ecological variations reflect the number of cases and deaths from schistosomiasis throughout the country [5,7,24]. Hereupon, the Northeast region still presents precarious indicators of drinking water supply and sewerage system, and in accessing to specialized health services, mainly in inland cities [6,25]. In addition to promoting the maintenance of the parasite's transmission cycle, these conditions cause delays in the diagnosis and timely treatment of ill patients. As a result, there is a higher risk for the occurrence of severe clinical forms, as the chronic hepatosplenic form, and death [12,26].

Remarkable, assessing comorbidities associated to schistosomiasis expands knowledge about clinical aspects and the epidemiological profile of the disease. Importantly, due to the increase in the number of cases in the elderly and the life expectancy among patients with schistosomiasis in recent decades, there was a greater likelihood of the coexistence of chronic diseases such as neoplasms, diabetes, obesity, and hypertensive diseases [26,27]. Correspondingly, we identified many disorders related to the digestive, circulatory, and respiratory systems, which may be indicative of the clinical complications of the chronic hepatosplenic phase of the disease [28,29].

Additionally, we observed a high number of infectious diseases as associated causes to schistosomiasis, mainly viral hepatitis, septicemia, and pneumonia. Prior studies have investigated the association between schistosomiasis and hepatitis caused by viruses B and C, especially in Egypt and Brazil [12,30-32]. The authors emphasize that the occurrence of both infections can further aggravate the patient's clinical condition, with a greater risk of liver fibrosis, portal hypertension, and the progression of hepatic fibrosis into cirrhosis and hepatocellular carcinoma, which considerably increases the risk of death [31,32].

In this study, the high number of deaths from schistosomiasis associated with hypertensive diseases and diabetes mellitus can be related to the increase in mortality in the elderly, where these coexisting diseases are more common [33]. Furthermore, the presence of renal and cardiovascular diseases can be even more severe in patients with the hepatosplenic chronic form of schistosomiasis [28,29]. Therefore, the high number of coexisting illnesses can be an indicator of a worse prognosis in elderly patients, and it assumes that there is a higher risk of death from schistosomiasis. Taken together, our findings reinforce the need to assess the occurrence of comorbidities in patients with schistosomiasis, especially in those with chronic forms, in order to initiate appropriate treatment and reduce the risk of clinical complications and death.

Unexpectedly, we identified death related to S. haematobium or S. japonicum infection, and other schistosomiasis, which may include the species S. *intercalatum*, S. *mattheei*, and S. *mekongi*. Notwithstanding, these Schistosoma species are commonly found in Africa and Asia [1]. Considering that Brazil does not have records of snails for the maintenance of the cycle of these species [5], it is unlikely that autochthonous transmission of other species of Schistosoma occurs in the country. Thereby, our findings may be due to the presence of Brazilians who traveled to endemic areas, became infected, and returned to Brazil where the death occurred. Alternatively, these data may be due to the death of immigrants or refugees from these endemic areas, which has increased exponentially in recent decades in Brazil [34,35].

The spatial distribution maps showed that municipalities with deaths from schistosomiasis are located mostly in the coastal strip of the Northeast and Southeast regions. In addition, these areas had high number of deaths caused by non-endemic *Schistosoma* species in Brazil^[^36^]^. Notably, states from the Northeast and Southeast regions have become the highest areas of flow of tourists and of immigrants in Brazil, mainly in the municipalities of the coastal areas. Importantly, the high number of visitors exposed to the *S. mansoni* infection in these areas may contribute to the disease spreading across the country and even to other continents.

Interestingly, most municipalities in Brazil had unspecified schistosomiasis as the leading cause of death, and not schistosomiasis caused by *S. mansoni*, as expected. These findings may indicate lacks in the accurate diagnosis of the disease's etiology, as underreporting or misdiagnosis, or due to the inadequate filling out of death certificates by medical professionals. Nevertheless, the still significant number of deaths from schistosomiasis, historically endemic in the country, demonstrate failures in the disease control actions, as timely diagnosis and treatment to reduce the clinical complications of the disease [37].

Prior studies have reported a reduction in the number of *S. mansoni* infection in Brazil and suggesting the efficiency of PCE activities for controlling the disease [7,22]. Conversely, a study conducted by Cruz and colleagues [38] in the state of Sergipe, Northeastern Brazil, demonstrated failures in the conduct of the PCE in the state. Regardless the drop in the positivity rate from 2008 to 2017 (10.3% to 7.1%), there was also a significant reduction in the number of municipalities carrying out the PCE activities (APC = -3.96) and in the number of parasitological tests performed (APC = -9.58). The authors concluded that there was a decrease in the actions of the PCE in the municipalities of Sergipe. Furthermore, there was a tendency to increase cases with high parasitic load and, probably, severe cases. Thereby, we speculate that failures in the PCE activities may also be occurring in other states, which may explain the stable trend of deaths from the disease in Brazil.

Currently, the main effective actions to control schistosomiasis in endemic areas include diagnosis, mass treatment with the anthelminthic drug praziquantel, and *Biomphalaria* snails control [4,39,40]. Meanwhile, considering that schistosomiasis is a neglected tropical disease, we suggest that the goal of elimination schistosomiasis as a public health problem by 2030, as established by the WHO, will only be achieved if, in addition to those measures, there are improvements in the sewerage system and adequate water supply in social vulnerable areas in Brazil [1,6]. Despite this, in virtue of coronavirus disease 2019 (COVID-19), many diagnosis and mass treatment campaigns for schistosomiasis have been halted, with uncertain implications for the programs [40].

Regardless of these findings, our study has some limitations that deserve to be mentioned. Considering that we used secondary and public domain data, mortality rates can still be underreported, even after the notable progress made with improvements in the systems for recording deaths by SIM in Brazil. Additionally, the lack of PCE's actions in some municipalities may limit the spatial data analysis. Finally, many chronic diseases affect mostly the elderly population, therefore, future studies should implement techniques to evaluate groups of cases while simultaneously adjusting the age range and/or other relevant covariates.

In the light of the above, our findings reinforce the importance of schistosomiasis as a serious and persistent public health concern in Brazil. Despite the implementation of the PCE control actions in the last decades, the disease showed a stable time trend of deaths in Brazil, among women, and in elderly. Furthermore, there was increasing trend in the Northeast region. Additionally, the occurrence of deaths caused by non-endemic *Schistosoma* species in Brazil reinforces the need for laboratory evaluation and clinical care in immigrants and refugees, and for Brazilians who have visited endemic areas for schistosomiasis in other countries. Considering this, improvements in the PCE actions, along with investments in public policies for education, public health, and the social inequalities in Brazil are essential to disrupt the biological cycle of the disease and, therefore, to reduce the schistosomiasis transmission and deaths, especially in low-income areas.

## Additional material


Online Supplementary Document
