# Supplementary material for: Basic and associated causes of schistosomiasis-related mortality in Brazil: A population-based study and a 20-year time series of a disease still neglected
Source: J Glob Health. 2021 Oct 9;11:04061. doi: 10.7189/jogh.11.04061 (PMC8542380; doi:10.7189/jogh.11.04061)
Supplement: Online Supplementary Document [file jogh-11-04061-s001.pdf]

Table S1. Ranking of the states of Brazil, according to the number of deaths from schistosomiasis, as an associated cause, and schistosomiasis mortality rates per 100,000 inhabitants, between 1999 to 2018.

**States of Brazil**	**Schistosomiasis-related deaths**		**Mortality rate per 100,000 inhabitants**
	**(n)**	**(%)**	
Pernambuco	1,637	39.28	0.933
São Paulo	786	18.86	0.095
Alagoas	483	11.59	0.769
Minas Gerais	450	10.80	0.113
Bahia	319	7.65	0.109
Sergipe	106	2.54	0.264
Rio de Janeiro	101	2.42	0.032
Espírito Santo	64	1.54	0.092
Paraíba	58	1.39	0.077
Ceará	30	0.72	0.018
Distrito Federal	24	0.58	0.046
Rio Grande do Norte	22	0.53	0.035
Paraná	19	0.46	0.009
Maranhão	17	0.41	0.013
Goiás	15	0.36	0.013
Rondônia	12	0.29	0.040
Pará	7	0.17	0.005
Mato Grosso	5	0.12	0.008
Rio Grande do Sul	4	0.10	0.002
Mato Grosso do Sul	3	0.07	0.012
Tocantins	3	0.07	0.006
Santa Catarina	1	0.02	0.001
Piauí	1	0.02	0.002
Amazonas	1	0.02	0.001
Acre	0	0.00	0
Roraima	0	0.00	0
Amapá	0	0.00	0
